# Comprehensive Decision Tree Models in Bioinformatics

**DOI:** 10.1371/journal.pone.0033812

**Published:** 2012-03-30

**Authors:** Gregor Stiglic, Simon Kocbek, Igor Pernek, Peter Kokol

**Affiliations:** 1 Faculty of Health Sciences, University of Maribor, Maribor, Slovenia; 2 Faculty of Electrical Engineering and Computer Science, University of Maribor, Maribor, Slovenia; American University in Cairo, Egypt

## Abstract

**Purpose:**

Classification is an important and widely used machine learning technique in bioinformatics. Researchers and other end-users of machine learning software often prefer to work with comprehensible models where knowledge extraction and explanation of reasoning behind the classification model are possible.

**Methods:**

This paper presents an extension to an existing machine learning environment and a study on visual tuning of decision tree classifiers. The motivation for this research comes from the need to build effective and easily interpretable decision tree models by so called one-button data mining approach where no parameter tuning is needed. To avoid bias in classification, no classification performance measure is used during the tuning of the model that is constrained exclusively by the dimensions of the produced decision tree.

**Results:**

The proposed visual tuning of decision trees was evaluated on 40 datasets containing classical machine learning problems and 31 datasets from the field of bioinformatics. Although we did not expected significant differences in classification performance, the results demonstrate a significant increase of accuracy in less complex visually tuned decision trees. In contrast to classical machine learning benchmarking datasets, we observe higher accuracy gains in bioinformatics datasets. Additionally, a user study was carried out to confirm the assumption that the tree tuning times are significantly lower for the proposed method in comparison to manual tuning of the decision tree.

**Conclusions:**

The empirical results demonstrate that by building simple models constrained by predefined visual boundaries, one not only achieves good comprehensibility, but also very good classification performance that does not differ from usually more complex models built using default settings of the classical decision tree algorithm. In addition, our study demonstrates the suitability of visually tuned decision trees for datasets with binary class attributes and a high number of possibly redundant attributes that are very common in bioinformatics.

## Introduction

Decision trees are one of the most popular classification techniques in data mining [1]. One of the main reasons for this is decision trees' ability to represent the results in a simple decision tree format which is easy to interpret for experts, as they can see the structure of decisions in the classifying process. The basic idea of the decision tree format is to construct a tree whose leaves are labeled with a particular value for the class attribute and whose inner nodes represent descriptive attributes. Given an inner node N, the children of N correspond to different possible values of the associated descriptive attribute. Once a decision tree is built, determining the class value for a new instance is achieved by following a path from the root to a leaf according to the values of the descriptive attributes of the instance. The class value assigned will be that labeling the leaf. Following this process one can easily extract classification rules that can be readily be expressed so that humans can understand them. In addition to their simplicity, building decision trees is often a less time consuming classification process compared to other classification techniques [2], and decision tree rules can be directly used as statements in a database access language (e.g. SQL).

Decision trees can be built with several different approaches where the most popular are C4.5 [3] and CART [4]. Due to their popularity, decision trees have been applied to different research fields including bioinformatics [5], [6], medicine [7] and image classification [8]. In addition, several commercial products use decision trees for knowledge discovery, predictive analysis and other purposes. For instance, KnowledgeSeeker [9] offers business intelligence software for customer analytics and marketing analytics.

From the knowledge discovery perspective, the ability to track and evaluate every step in the decision-making process is one of the most important factors for trusting the decisions gained from data-mining methods. Examples of such techniques are decision trees that possess an important advantage in comparison with competitive classification methods - i.e., the symbolic representation of the extracted knowledge. Decision trees, along with rule-based classifiers, represent a group of classifiers that perform classification by a sequence of simple, easy-to-understand tests whose semantics are intuitively clear to domain experts [10]. Although current state-of-the art classifiers (e.g. Support Vector Machines [11]) or ensembles of classifiers (e.g. Random Forest [12] or Rotation Forest [13]) significantly outperform classical decision tree classification models in terms of classification accuracy or other classification performance metrics, they are not suitable for knowledge discovery process.

When decision trees are used in knowledge discovery, one should usually include domain experts in the analysis process. Therefore, in most cases the final decision trees will be presented to domain experts for evaluation of extracted knowledge – i.e., rules that can be derived from a decision tree. In such cases the complexity of decision trees, which is usually measured as the number of nodes or the number of rules that can be extracted from a tree, is of high importance and can influence the evaluation of the discovered knowledge by domain experts [14]. Decision tree complexity has been studied in terms of reducing the complexity and maintaining or improving the accuracy at the same time. Bohanec and Bratko [15] studied the difference between pruning a decision tree for better approximation of the target concept and pruning the decision tree to make it practical for communication and understanding by the user. Their study focused on developing algorithms for obtaining the smallest pruned decision trees that represent concepts within some chosen accuracy. Oates and Jensen [16] studied the influence of database size on decision tree complexity. They demonstrated that the tree size strongly depends on the training set size. Therefore, many approaches that are based on removing training instances prior to tree construction [17], [18], [19] could result in smaller trees just because of the training set reduction.

Different visual representations of decision trees like the classical node-link diagrams [20], [21], treemaps [22], [23], concentric circles [24], [25], and many others have been proposed in the past. A major consideration in evaluation of decision trees is also how efficiently they use screen space to communicate the tree information [26]. Through application of decision trees to different fields of research and their use in open source and commercial software for machine learning and data mining, it has been demonstrated that end-users still prefer node-link diagrams although their space covering is not optimal. Huysmans et al. [27] observe that currently, most research focuses on improving the accuracy or precision of predictive models and comparatively little research has been undertaken to increase their comprehensibility to the analyst or end-user. They empirically investigated suitability of decision tables, (binary) decision trees, propositional rules, and oblique rules in environments where interpretability of models is of high importance. The results showed that users prefer decision tables, followed by decision trees to other compared knowledge representations, but authors admitted that only inexperienced users were included in the study.

A multi-criteria approach to evaluation of decision trees that also includes size of the built decision trees was proposed by Osei-Bryson [28]. It aims to make the data mining process simpler for data mining project teams, especially when they have to evaluate significant number of decision trees. The proposed project uses three measures to evaluate appropriateness of the decision trees: stability, simplicity and discriminatory power. Simplicity, or equivalently complexity is further divided in the number of rules that can be extracted from the tree and the average length of the extracted rules.

Due to their popularity and a need to build simple decision trees with as little effort as possible, this paper proposes a novel method called Visual Tuning of Decision Trees (VTDT). This method helps data analysts in building effective decision tree representations with spending less time on setting and tuning the parameters of decision tree induction algorithm when compared to classical methods. From the analyst's perspective it is very important that the produced representation of the decision tree allows effective communication with end-users (i.e. customers) or domain experts in cases of decision tree applications in research. In addition, from our own and experience of our colleagues, we know that, although we live in a digital age, we still meet a lot of experts in different domains who prefer to have the final decision tree printed out on a sheet of paper. The result of the VTDT method is a decision tree that can be printed out on a single page or displayed on a computer screen without the need for scrolling or zooming. It is also important to take care of the decision trees that would be too pruned when using the default parameters of decision tree induction method. One could also call this type of decision tree induction “one-button decision trees” as there is no need to tune the parameters and build multiple decision trees anymore.

## Methods

The proposed method in this paper presents an automated tuning process for the widely used C4.5 decision tree, which was developed by Quinlan [3]. More precisely, it focuses on C4.5's implementation in the Weka machine learning framework [29], where it is referred to as J48.

### 2.1 Tuning the Parameters

There are multiple settings that can influence the size of the generated decision tree. Two types of pruning are available - i.e., subtree replacement and subtree raising. Subtree raising uses a technique where a node may be moved upwards towards the root of the tree, replacing other nodes along the way during a process of pruning. In general, subtree raising is computationally more complex than subtree replacement where the nodes in a decision tree can be replaced by leafs. Another setting influencing the pruning process is confidence factor that represents a threshold of allowed inherent error in data while pruning the decision tree. By lowering the threshold one is applying more pruning and consequently generates more general models. To obtain simpler models where leafs contain higher number of samples, it is possible to set the minimal number of objects in a single leaf. This setting can also be used in tuning to achieve simpler and smaller decision trees. The final setting that can be used to tune the visual outlook of the tree is called binary splits selection. This setting forces the splitting of nodes to only two branches instead of multiple splits. The default J48 decision tree in Weka uses pruning based on subtree raising, confidence factor of 0.25, minimal number of objects is set to 2, and nodes can have multiple splits. To allow automated tuning in Weka, a package called Visually Tuned J48 (VTJ48, available at http://ri.fzv.uni-mb.si/vtj48/) was developed during this study.

All parameters, mentioned in the previous paragraph, are automatically tuned in VTJ48 to allow the so called “one-button data mining”. However, it is possible to change the default values for dimensions of the resulting window that represent boundaries of the VTJ48 decision tree. Default values for maximal dimensions of the decision tree are set to 1280×800 pixels corresponding to the Widescreen eXtended Graphics Array (WXGA) video standard. The aspect ratio of this resolution is 16∶10 (1.60) and comes very close to aspect ratio of A4 paper dimensions (approx 1.41). The chosen dimensions can also be displayed on most computer monitors in use today.

Although it would be possible to use the original Weka source code to display decision trees, some adaptations to original decision tree visualization methods had to be done to allow better covering of space for nodes and leaves. In comparison to classical Weka decision tree visualization, we changed the shape of internal nodes to allow more space on both sides of nodes. Additionally, we reduced the height of the trees with reduction of the vertical distance between nodes by 50%.

**Figure 1:**
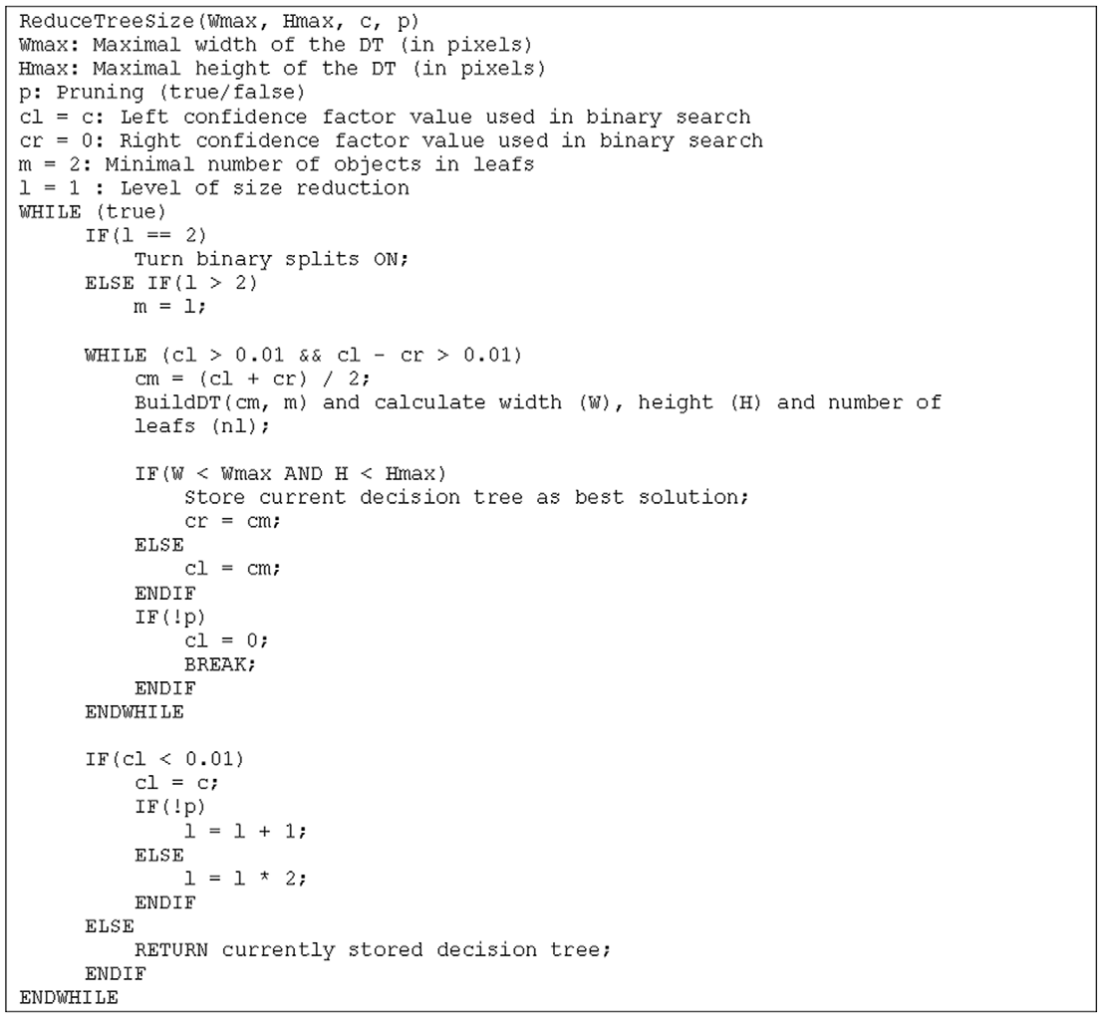
Comparison of the original J48 decision tree (upper image) and visually tuned version from VTJ48 (lower image) on the letter dataset.

Tuning of parameters in VTJ48 is done using adapted binary search where confidence factor of pruning is optimized until highest acceptable value of this parameter is found. Boundaries for confidence factor optimization are set at 0 and 0.5 (starting value in VTJ48 and the maximal allowed setting in J48). In cases where initial confidence factor tuning cannot build an acceptable decision tree, binary splits are turned on. This step usually significantly reduces horizontal dimensions of the tuned decision tree. Tuning of confidence factor is done once again. In rare cases, where binary splits are not enough, VTJ48 tries to increase minimal number of objects in leaves. This parameter (*m*) is increased from 2 until *m<n* in steps of *m^2^*, where *n* is number of all samples in the training set. More extensive search could have been chosen, but in such case one should expect a significant increase in the time complexity of the tuning process. The pseudocode in Figure 1 describes the reduction of the tree size process as implemented in VTJ48.

In rare cases the default settings of VTJ48 algorithm will produce an extremely small tree consisting of just one or even without splitting nodes. Therefore, in cases of decision trees with only one or two leaves, an approach using unpruned decision tree is used. With confidence factor set to 0.5 such tree will usually grow over the predefined boundaries. This time a linear hill-climbing approach is used to increase the minimal number of objects in leaves, because there is no need to tune confidence factor in an unpruned decision tree.

### 2.2 Experimental Settings

By reducing the size and complexity of decision trees to fit the predefined screen resolution or paper size one is expecting significantly lower classification accuracy, especially in initially very large decision trees. We used several different datasets to test this assumption.

#### 2.2.1. UCI Datasets

Forty UCI repository [30] datasets retrieved from the Weka website were used to evaluate the classification performance of the VTDTs. Basic information including the information on attributes that can influence the size of a decision tree for all datasets is presented in Table 1.

**Table 1:** Basic information on 40 datasets from UCI repository used in this study including information about number of instances, attributes, classes, length of longest attribute name (LAN) and length of the longest nominal attribute value (LAV).

Dataset	Samples	Attributes	Nominal	Numeric	Classes	LAN	LAV
anneal	898	39	33	6	6	22	5
anneal.orig	898	39	33	6	6	22	5
arrhythmia	452	280	74	206	16	28	2
audiology	226	70	70	0	24	23	32
autos	205	26	11	15	7	17	13
balance-scale	625	5	1	4	3	14	1
breast-cancer	286	10	10	0	2	11	20
breast-w	699	10	1	9	2	21	9
colic	368	23	16	7	2	27	29
colic.orig	368	28	21	7	2	27	7
credit-a	690	16	10	6	2	5	2
credit-g	1000	21	14	7	2	22	30
diabetes	768	9	1	8	2	5	15
ecoli	336	8	1	7	8	5	3
glass	214	10	1	9	7	4	20
heart-c	303	14	8	6	5	8	21
heart-h	294	14	8	6	5	10	21
heart-statlog	270	14	1	13	2	36	7
hepatitis	155	20	14	6	2	15	6
hypothyroid	3772	30	23	7	4	25	23
ionosphere	351	35	1	34	2	5	1
iris	150	5	1	4	3	11	15
kr-vs-kp	3196	37	37	0	2	5	5
labor	57	17	9	8	2	30	13
letter	20000	17	1	16	26	5	1
lymph	148	19	16	3	4	15	12
mushroom	8124	23	23	0	2	24	1
optdigits	5620	65	1	64	10	7	1
pendigits	10992	17	1	16	10	7	1
primary-tumor	339	18	18	0	22	15	17
segment	2310	20	1	19	7	20	9
sick	3772	30	23	7	2	25	8
sonar	208	61	1	60	2	12	4
soybean	683	36	36	0	19	15	27
splice	3190	62	62	0	3	13	24
vehicle	846	19	1	18	4	25	4
vote	435	17	17	0	2	38	10
vowel	990	14	4	10	11	14	6
waveform-5000	5000	41	1	40	3	5	1
zoo	101	18	17	1	7	8	12

#### 2.2.2. Protein Solubility Datasets

In addition to the datasets from the UCI repository, we tested our method on datasets in the field of bioinformatics.

Protein solubility is an important protein property since low protein solubility can lead to several diseases [31] or affect isolation of proteins from complex mixtures [32]. Several attempts to classify and predict protein solubility have been made [33]–[36]. To assess our method, we used the eSol database (available at http://tp-esol.genes.nig.ac.jp/) which includes information about protein solubility of the entire ensemble of E.coli proteins. The database contains 1,625 proteins, out of which 782 are insoluble and 843 are soluble proteins. We calculated 21 feature datasets for each of these proteins as shown in Table 2. These numeric features have shown to be influential in protein solubility prediction in previous works, where:

the feature datasets 1–18 contain mono-, di- and tri-mers using 7 different alphabets,
the feature dataset 19 contains 4 sequence-computed features, i.e., molecular weight, sequence length, isolectric point and GRAVY index,
the feature dataset 20 contains features used in [33], and
the feature dataset 21 combines all features from the previous datasets.

**Table 2:** Feature datasets used in protein solubility classification.

#	Name	Size
1	MonomersNatural	20
2	DimersNatural	13
3	TrimersNatural	24
4	MonomersHydro	5
5	TrimersHydro	12
6	MonomersConfSimi	7
7	DimersConfSimi	20
8	TrimersConfSimi	15
9	MonomersBlosum	8
10	DimersBlosum	25
11	MonomersClustEm14	14
12	DimersClustEm14	16
13	TrimersClustEm14	22
14	MonomersClustEm17	17
15	DimersClustEm17	27
16	TrimersClustEm17	42
17	MonomersPhysChem	7
18	DimersPhysChem	21
19	Computed	4
20	eSol	22
21	All Features	342

#### 2.2.3. Gene Expression Datasets

Comprehensible classifiers can provide an important insight in gene expression analysis studies. In this study we used 9 Gene Expression Machine Learning Repository (GEMLeR) datasets [37]. Altogether 1545 samples are divided in the following groups by tumor type: breast (344 samples), colon (286), kidney (260), ovary (198), lung (126), uterus (124), omentum (77), prostate (69) and endometrium (61). GEMLeR datasets used in this study were created by selecting one out of 9 groups of samples in so called one-versus-all binary classification setting. Unsupervised highest variance filter was chosen to avoid the so called “selection bias” when reducing the number of attributes by eliminating the measurements with extremely low variance. Samples consisting of original 54,681 expression measurements from Human Genome U133 Plus 2.0 Array GeneChip were reduced to 10,935 (20%) gene expression measurements that represent attributes of 9 datasets.

#### 2.2.4. Performance Evaluation

Different measures were observed for J48 decision tree using default settings and VTJ48 decision tree on all datasets. Basic size related measures like width and height of decision tree in pixels, number of leaves and number of nodes were calculated for each decision tree on each dataset. Additionally, Classification accuracy (ACC) and area under ROC curve (AUC) were calculated using 20 runs of 10-fold cross-validation on all datasets to observe differences in classification performance.

## Results

To evaluate the proposed method we compared the classification performance and size of the classical C4.5 trees (J48) with the visually tuned C4.5 trees (VTJ48). Initially, the tests were performed on 40 datasets from the well-known machine learning repository. In addition, the tests were done on two types of datasets where decision trees can be applied in the field of bioinformatics - i.e., 21 protein solubility datasets and 9 gene expression analysis datasets.

As expected, in most cases, the original J48 decision tree vastly exceeded the predefined display resolution of 1280×800 pixels (Table 3 and Table 4). In some extreme cases the width of the decision tree exceeded the predefined dimension by more than 10-fold (letter, audiology, soybean). However, decision trees of this size and high number of classes are inappropriate for extraction of rules and presentation to end-user. Altogether, in the UCI datasets evaluation, there are 30 datasets where VTJ48 optimized a decision tree by reducing the number of leaves to fit into predefined dimensions. In 8 cases VTJ48 produced decision trees with more leaves than the original J48 method. Increase of the tree size occurred in cases when there were only one or two leaves produced using default settings of J48, pruning was automatically turned off in VTJ48 resulting in more complex decision trees. In case of protein solubility datasets, there were 20 datasets where the complexity of the tree was reduced and only one case where it increased. Similar changes in tree complexity were observed in gene expression problems, where complexity increased only in 2 out of 10 datasets. Observing the complexity of built decision trees one should also note that VTJ48 starts the tuning process of confidence factor at 0.5, whereas J48 starts at 0.25 resulting in more complex VTJ48 decision trees that still fit into predefined visual boundaries.

**Table 3:** Comparison of decision tree dimensions on 40 UCI datasets including the number of leaves.

	Leaves		Width		Height	
	J48	VTJ48	J48	VTJ48	J48	VTJ48
anneal	37.69	12.98	2753.62	2555.43	670.11	677.68
anneal.ORIG	46.37	11.10	3426.41	1362.30	868.05	546.30
arrhythmia	40.59	10.20	1679.34	1589.04	1555.57	1462.47
audiology	30.25	9.11	3799.18	3781.91	923.98	921.00
autos	45.25	12.77	6527.37	4199.37	654.02	637.58
balance-scale	41.24	25.86	1986.12	1222.98	821.96	747.91
breast-cancer	9.60	4.04	1177.92	1518.04	348.63	354.23
breast-w	12.08	14.23	781.99	967.01	637.84	698.75
colic	6.07	8.76	546.44	1198.39	360.41	424.61
colic.ORIG	1.00	6.83	1.00	480.83	1.00	372.96
credit-a	21.40	12.01	1664.81	1098.50	669.91	619.21
credit-g	89.05	7.07	13906.86	1077.07	877.89	335.60
diabetes	21.87	11.97	1488.65	963.30	830.31	694.87
ecoli	18.70	17.78	1039.47	1055.72	735.37	723.22
glass	23.73	12.23	2293.10	1838.96	827.88	754.41
heart-c	26.05	8.85	3273.48	1399.74	618.49	476.47
heart-h	7.21	8.17	673.28	1042.54	408.37	464.92
heart-statlog	17.85	13.41	1577.55	1309.66	633.84	605.13
hepatitis	9.24	12.41	522.66	754.78	571.69	659.98
hypothyroid	14.39	13.43	1101.64	1070.54	756.02	771.15
ionosphere	13.85	11.59	1070.98	1019.02	775.47	734.02
iris	4.69	4.76	227.87	231.10	428.43	432.21
kr-vs-kp	28.98	13.16	1187.64	1104.25	1091.28	1076.77
labor	4.00	5.20	329.00	464.17	333.06	380.56
letter	1165.00	12.65	63285.55	63344.28	1916.69	1919.54
lymph	17.43	10.12	1863.97	1252.03	580.12	462.35
mushroom	24.93	24.93	1022.25	1022.25	527.00	527.00
optdigits	205.46	16.09	11154.56	11195.65	1330.36	1334.04
pendigits	188.13	16.04	10719.41	10784.98	1297.69	1296.05
primary-tumor	43.18	14.62	3794.33	1797.86	891.43	789.16
segment	41.12	11.09	3749.02	3748.84	1084.95	1085.78
sick	27.59	14.22	1763.57	1087.54	815.68	710.67
sonar	14.71	13.80	1107.13	1089.68	665.59	659.63
soybean	61.28	11.04	6175.62	6180.02	913.67	920.67
splice	173.83	20.78	7537.58	6176.44	759.48	731.51
vehicle	69.22	16.27	5069.70	4183.99	1168.31	1065.60
vote	5.81	6.22	390.94	432.98	508.98	513.86
vowel	126.41	10.58	11046.43	11045.28	985.60	986.01
waveform-5000	295.66	16.82	16325.97	13756.92	1494.51	1386.66
zoo	8.31	8.31	436.69	436.69	567.50	567.50

**Table 4:** Comparison of decision tree dimensions on the protein feature datasets including the number of leaves.

	Leaves		Width		Height	
	J48	VTJ48	J48	VTJ48	J48	VTJ48
MonomersNatural	91.73	13.08	6965.05	4779.43	1296.91	1147.77
DimersNatural	54.05	12.93	3880.20	1807.83	1226.37	882.51
TrimersNatural	15.57	11.03	1403.25	1338.10	798.08	784.64
MonomersHydro	7.25	7.05	576.10	600.18	547.75	553.87
TrimersHydro	41.54	11.38	3035.91	1498.45	1068.53	816.91
MonomersConfSimi	16.55	11.91	1225.22	999.07	765.66	701.04
DimersConfSimi	85.58	13.02	6518.23	3880.32	1256.65	1045.51
TrimersConfSimi	37.49	11.02	2607.05	1251.43	1112.98	807.38
MonomersBlosum	29.72	13.61	2270.26	1399.86	909.40	781.34
DimersBlosum	94.21	13.27	7139.47	4640.00	1297.53	1129.44
MonomersClustEm14	68.44	13.46	5272.88	3006.28	1169.90	984.28
DimersClustEm14	51.66	11.20	4115.81	1974.13	1202.74	921.91
TrimersClustEm14	35.04	10.53	2808.01	1310.34	1245.19	845.54
MonomersClustEm17	84.87	12.90	6687.42	3637.08	1182.30	956.30
DimersClustEm17	117.36	10.26	7419.49	3430.75	1609.62	1158.41
TrimersClustEm17	88.52	10.06	6730.81	3020.26	1912.71	1221.96
MonomersPhysChem	32.92	13.67	2655.87	1674.10	919.93	831.75
DimersPhysChem	80.22	10.81	5927.14	2879.09	1356.17	1047.01
Computed	7.99	8.51	724.04	779.70	516.48	534.92
eSol	89.79	14.13	7611.00	4331.36	1124.45	976.64
All Features	111.09	13.14	6949.88	4988.28	1744.36	1448.88

### 3.1 Classification Performance on UCI Data

Accuracy and AUC (Table 5) were used for evaluation of classification performance, although due to the high number of multiclass datasets, it is debatable whether accuracy is the right measure for classification performance. As suggested in [38], the Wilcoxon signed ranks test was used to assess statistical significance of difference in performance and complexity of the decision tree. Comparing accuracy using win/draw/lose record one can observe J48 wins on 21 datasets, while VTJ48 managed to outperform J48 on 16 datasets. Statistical significance testing shows that J48 significantly outperforms VTJ48 in accuracy (p = 0.022), while there is no significant difference in results of AUC (p = 0.766). As already mentioned, one should be cautious when interpreting the results above, since accuracy is not a well suited performance measure in cases of unbalanced multi-class datasets. Therefore we did another test where only 16 binary class datasets were used and found out that there are no statistically significant differences present (p = 0.320). Table 3 demonstrates a big difference in decision tree size (number of leaves) comparing J48 to VTJ48 decision trees.

**Table 5:** Comparison of classification performance (20 runs of 10-fold cross-validation) on 40 UCI datasets.

	Accuracy		AUC		Δ (J48 - VTJ48)	
	J48	VTJ48	J48	VTJ48	ACC	AUC
Anneal	98.64±0.2	98.93±0.2	99.36±0.3	98.85±0.3	−0.28	0.51
anneal.orig	92.34±0.5	81.34±1	97.47±0.4	83.6±2.6	11.00	13.87
arrhythmia	65.88±1.1	70.63±1	73.58±1.4	79.01±1	−4.75	−5.43
audiology	77.3±1.4	66.31±3.9	92.31±0.6	91.78±1	11.00	0.53
Autos	82.59±2.6	64.07±2.4	91.45±1.1	82.42±2.4	18.51	9.04
balance-scale	77.9±0.9	77.35±0.7	82.36±0.8	83.93±1.1	0.55	−1.57
breast-cancer	74.25±0.8	74.48±0.9	58.76±1.8	59.69±1.5	−0.23	−0.93
breast-w	94.64±0.4	94.69±0.4	95.21±1	95.44±0.6	−0.06	−0.23
Colic	85.15±0.4	85.03±0.7	80.79±0.9	81.12±1.2	0.12	−0.33
colic.orig	66.3±0	65.33±1.7	48.55±0	70.31±1.5	0.98	−21.76
credit-a	85.83±0.7	86.24±0.7	88.49±0.7	89.18±0.8	−0.41	−0.70
credit-g	71.03±0.8	71.85±0.6	64.46±1.2	70.96±0.6	−0.82	−6.50
diabetes	74.29±1.1	74.52±1.1	75.31±1.3	74.6±1.4	−0.23	0.71
Ecoli	82.96±1.2	82.62±1.1	90.63±0.8	91.03±0.6	0.34	−0.40
Glass	67.17±2.5	67.78±2.2	80.13±2	80.97±1.3	−0.61	−0.85
heart-c	76.85±1.6	76.2±1.6	77.24±2.4	77.73±2	0.64	−0.49
heart-h	78.33±1.1	78.4±1.2	75.22±1.5	77.53±1.8	−0.07	−2.31
heart-statlog	77.83±1.7	78.56±2.1	77.49±2.6	77.91±2.2	−0.72	−0.42
hepatitis	79.77±1.9	79.84±1.7	67.57±4.6	70.54±4.7	−0.06	−2.97
hypothyroid	99.53±0	99.55±0	99.27±0.2	99.28±0.2	−0.02	−0.01
ionosphere	89.9±1.1	89.93±1	88.95±1.7	88.11±1.4	−0.03	0.83
Iris	94.7±0.9	94.7±0.9	95.73±0.7	95.76±0.8	0.00	−0.03
kr-vs-kp	99.39±0.1	97.35±0.1	99.81±0	99.41±0.1	2.03	0.40
Labor	80.09±3.1	82.28±3.1	72.05±4.7	75.89±4.4	−2.19	−3.85
Letter	88.02±0.2	29.42±0.6	95.4±0.1	88.77±0.2	58.60	6.63
Lymph	77.03±1.5	76.45±1.9	79.39±1.9	78.73±3	0.57	0.66
mushroom	100±0	100±0	100±0	100±0	0.00	0.00
optdigits	90.51±0.2	73.94±0.4	95.39±0.1	93.69±0.1	16.57	1.70
pendigits	96.53±0.1	80.13±0.6	98.44±0.1	96.89±0.1	16.40	1.56
primary-tumor	42.68±1.5	41.83±1	71.95±0.8	71.52±1.1	0.86	0.43
segment	96.93±0.2	92±0.3	98.66±0.1	98.34±0.1	4.92	0.32
Sick	98.73±0.1	98.38±0.1	95.51±0.7	92.05±1.1	0.35	3.46
Sonar	72.07±3.1	72.26±2.6	73.58±3.3	73.13±3.2	−0.19	0.44
soybean	91.96±0.8	61.46±0.9	98.11±0.3	94.87±0.2	30.50	3.23
Splice	94.13±0.2	94.45±0.2	96.67±0.1	97.92±0.1	−0.32	−1.25
Vehicle	72.21±1.2	71.64±1	85.38±0.7	89.31±0.4	0.57	−3.93
Vote	96.41±0.4	96.38±0.4	96.97±0.4	97.03±0.4	0.03	−0.06
Vowel	80.11±1.3	43.39±1.1	92.34±0.6	87.78±0.5	36.72	4.56
waveform-5000	75.36±0.6	74.11±0.4	82.82±0.5	88.72±0.2	1.25	−5.90
Zoo	92.23±0.4	92.23±0.4	97.67±0.1	97.67±0.1	0.00	0.00
J48/tie/VTJ48	(21/3/16)		(17/2/21)			

### 3.2 Classification Performance on Bioinformatics Data

Table 4 shows the average decision tree dimensions for the protein datasets including the average number of leaves. It can be noticed that the size was reduced on the majority of the feature datasets. The only exceptions are the DimersClustEm14 and TrimersHydro datasets, on which the tree size increased.

Table 6 shows accuracy and AUC for the evaluation of classification performance on protein datasets. Since all these datasets present a binary classification problem, accuracy and ACC are more appropriate measurements when compared to the UCI datasets. Again, the Wilcoxon signed ranks test was used to assess statistical significance of difference in performance and complexity of the decision tree. When observing the accuracy win/draw/lose record, one can notice that J48 wins on 5 datasets, while VTJ48 managed to outperform J48 on 15 datasets. The results are similar for AUC where J48 wins on 5 datasets, while VTJ48 wins on 16 datasets.

**Table 6:** Comparison of classification performance (20 runs of 10-fold cross-validation) on the protein datasets.

	Accuracy		AUC		Δ (J48 - VTJ48)	
	J48	VTJ48	J48	VTJ48	ACC	AUC
MonomersNatural	70.76±0.9	72.41±0.7	70.58±1.3	76.41±0.6	−1.66	−5.83
DimersNatural	62.58±1	61.94±0.9	64.65±1	64.45±1	0.64	0.20
TrimersNatural	55.44±0.3	55.33±0.4	53.91±0.6	53.97±0.7	0.10	−0.06
MonomersHydro	64.64±0.9	64.58±0.9	68.1±0.8	68.08±0.8	0.06	0.02
TrimersHydro	62.79±0.8	63.25±0.6	64.43±1	64.68±0.7	−0.46	−0.25
MonomersConfSimi	66.75±0.9	66.79±0.9	71.97±0.9	71.96±0.8	−0.03	0.01
DimersConfSimi	64.68±1	66.51±0.6	63.35±1.1	68.82±0.9	−1.83	−5.48
TrimersConfSimi	63.25±0.8	63.69±0.7	65.77±1.1	66.28±0.8	−0.44	−0.51
MonomersBlosum	66.46±0.7	66.62±0.7	69.33±0.8	69.79±0.7	−0.16	−0.46
DimersBlosum	66.32±1	69.27±0.8	65.65±1.2	73.3±0.8	−2.95	−7.66
MonomersClustEm14	70.07±1	71.13±0.8	70.73±1	74.19±0.6	−1.06	−3.45
DimersClustEm14	66.87±0.8	67.52±1	69.33±1	71.23±0.9	−0.64	−1.90
TrimersClustEm14	73.74±0.7	76.31±0.7	73.62±1	80.43±0.8	−2.57	−6.81
MonomersClustEm17	72.69±0.8	74.22±0.5	72.44±1.2	77.12±0.6	−1.53	−4.68
DimersClustEm17	63.88±1	65.06±0.9	63.68±1	67.51±0.9	−1.18	−3.83
TrimersClustEm17	62.35±1	61.37±1.2	62.92±1.2	62.57±1.3	0.98	0.35
MonomersPhysChem	71.64±0.9	71.64±0.6	75.07±0.8	75.19±0.7	0.00	−0.12
DimersPhysChem	68.93±0.8	71.29±0.8	68.78±1.3	73.44±0.9	−2.36	−4.66
Computed	74.92±0.5	74.75±0.6	79.2±0.6	79.41±0.6	0.17	−0.21
eSol	61.16±0.8	61.47±0.8	63.67±0.9	63.6±0.9	−0.31	0.07
All Features	72.19±1	75.87±0.8	71.63±1.4	81.21±0.6	−3.68	−9.57
J48/tie/VTJ48	(5/1/15)		(5/0/16)			

Table 7 shows the average decision tree dimensions for the 9 GEMLeR datasets including the average number of leaves. In comparison to protein and UCI datasets it is evident that gene expression problems do not create very large tree, therefore the reduction in size, when VTJ48 is used is not that big.

**Table 7:** Comparison of decision tree dimensions on the GEMLeR datasets including the number of leaves.

	Leaves		Width		Height	
	J48	VTJ48	J48	VTJ48	J48	VTJ48
OVA_Breast	21.60	13.50	1673.00	1199.40	728.80	609.00
OVA_Colon	16.70	12.30	1608.30	1430.00	609.30	571.90
OVA_Endometrium	13.20	13.00	1129.50	1151.40	616.80	616.80
OVA_Kidney	11.50	11.10	1169.50	1117.90	542.00	549.50
OVA_Lung	12.00	13.20	1053.40	1069.70	616.60	661.20
OVA_Omentum	17.70	12.70	1291.30	1326.10	802.80	802.80
OVA_Ovary	25.50	13.90	2148.40	1842.00	773.20	743.40
OVA_Prostate	2.00	3.60	191.00	249.40	224.00	345.60
OVA_Uterus	23.60	15.30	1883.20	1563.80	758.50	721.50
OVA_Uterus	21.60	13.50	1673.00	1199.40	728.80	609.00

Table 8 shows accuracy and AUC for the evaluation of classification performance for the GEMLeR datasets and also demonstrates that the performance actually increases if we use simpler (i.e., smaller) decision tree models.

**Table 8:** Comparison of classification performance (20 runs of 10-fold cross-validation) on the GEMLeR datasets.

	Accuracy		AUC		Δ (J48 - VTJ48)	
	J48	VTJ48	J48	VTJ48	ACC	AUC
OVA_Breast	93.53±0.4	94.63±0.4	89.94±0.8	90.02±1	−1.10	−0.07
OVA_Colon	96.31±0.4	96.7±0.3	92.39±1.2	91.76±1.3	−0.39	0.62
OVA_Endometrium	95.15±0.4	95.08±0.5	63.57±6.5	64.11±5.4	0.06	−0.53
OVA_Kidney	96.38±0.3	96.31±0.3	93.03±0.8	93.25±0.7	0.06	−0.22
OVA_Lung	97.35±0.2	97.28±0.3	90.12±1.7	89.87±1.4	0.06	0.25
OVA_Omentum	93.98±0.5	94.43±0.4	54.82±5.9	67.99±7.9	−0.45	−13.16
OVA_Ovary	92.23±0.6	92.62±0.6	79.21±2.2	81.84±2.2	−0.39	−2.63
OVA_Prostate	99.68±0.1	99.61±0.1	97.02±1	98.69±0.8	0.06	−1.67
OVA_Uterus	92.17±0.4	92.43±0.3	73.16±3.5	70.22±3.2	−0.26	2.93
J48/tie/VTJ48	(4/0/5)		(3/0/6)			

Statistical significance testing was done on all 20 cross-validation run results for 30 bioinformatics datasets together using Wilcoxon signed-rank test. In case of accuracy (p = 0.002) and AUC (p = 0.001) the VTJ48 trees significantly outperformed J48 trees. Although we did not expect such significant differences between results in favor of VTJ48, it is obvious that VTJ48 is well suited for datasets with binary class attributes and a high number of possibly redundant attributes.

### 3.3 Examples of Large Decision Trees

**Figure 2:**
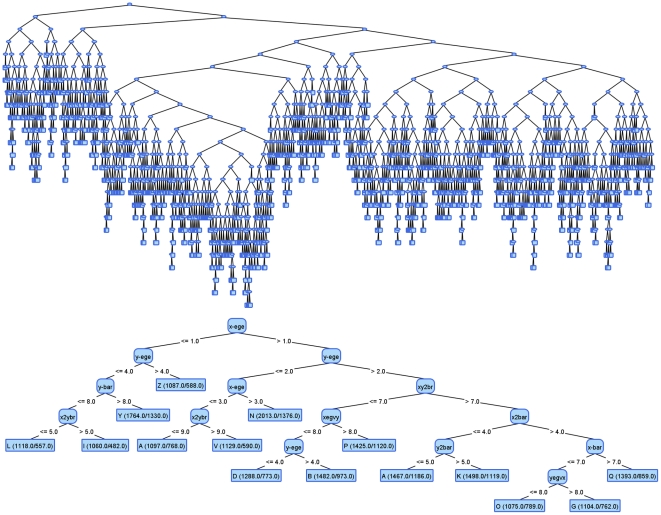
Comparison of original J48 decision tree and visually tuned version from VTJ48 on All Features dataset.

In this section we demonstrate two examples, each of them with two decision trees built on a single dataset. The first tree in each example is the result of J48 using default settings, and the second tree is the result of VTJ48.The dataset in the first example is the letter dataset from the UCI repository. This dataset contains 26 class values which represent 26 capital letters in the English alphabet. The character images were based on 20 different fonts and each letter within these 20 fonts was randomly distorted to produce 20.000 instances with 16 attributes. Fig. 2 shows the original tree and the visually tuned tree. One can notice the extremely complex original decision tree, which is the result of the high number of classes. Since the visually tuned tree does not cover all the possible classes, it cannot achieve competitive classification accuracy.

**Figure 3:**
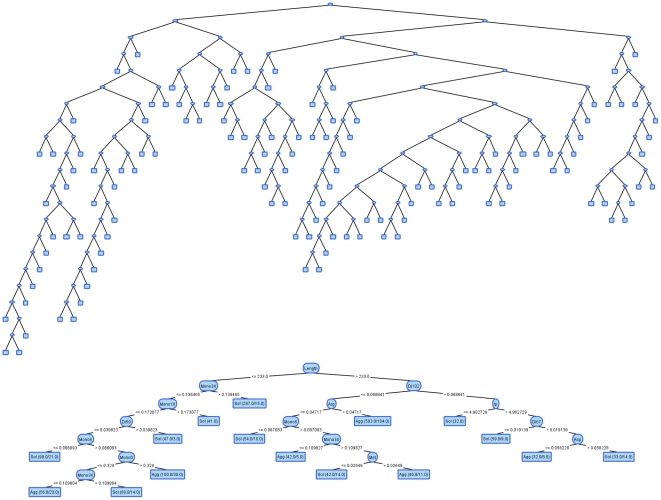
Comparison of durations for different datasets.

The second example presents two decision trees built on the protein solubility dataset with all features (Figure 3). In this case the accuracy and AUC were both improved significantly, when the size of the decision tree model was reduced. This is possible due to binary class attribute that still allows effective trees that are much smaller than the original pruned J48 trees.

In addition, to demonstrate the most significant rules from both decision trees in Figure 3, we extracted top 5 rules according to their support in training set (Table 9). The numbers at the end of mono-, di- and tri-mer attribute names (e.g. the number 34 in MonomersClustEm17_34) distinguish attributes inside different alphabets. It can be observed that J48 produces more complex rules with a higher number of conditions which use attributes from more different alphabets. On the other hand, top 5 rules from VTJ48 tree cover much more samples (70.6%) than top 5 rules derived from J48 (36.6%). It is evident that low error rates on the training set do not guarantee good classification performance on the test set. We can once again conclude that in most cases, at least in protein solubility domain, more complex trees result in overfitting to training samples.

**Table 9:** Top 5 rules with the highest support in All Features extracted from J48 and VTJ48 decision trees.

Rule	Conditions	Support	Error
**J48**			
IF Length< = 233 AND MonomersClustEm17_34>0.136 AND TrimersConfSimi_40< = 0.002 AND TrimersClustEm17_98< = 0.005 AND DimersConfSimi_19< = 0.069 THEN Soluble	5	228	1.32
IF Length>233 AND DimersClustEm17_102< = 0.069 AND MonomersNatural_0>0.047 AND Ip >5.181 AND TrimersClustEm17_96< = 0.002 AND Length >251 AND MonomersBlosum_14>0.074 AND TrimersNatural_19< = 0 AND MonomersNatural_1>0.039 THEN Insoluble	9	218	0.92
IF Length>233 AND DimersClustEm17_102< = 0.069 AND MonomersNatural_0< = 0.047 AND DimersClustEm14_70< = 0.002 AND TrimersClustEm17_90< = 0 AND DimersBlosum_40< = 0.015 AND MonomersClustEm14_20>0.132 AND TrimersClustEm17_85< = 0.003 THEN Soluble	8	53	5.66
IF Length>233 AND DimersClustEm17_102>0.069 AND DimersClustEm17_95< = 0.0121 AND DimersClustEm14_62>0.004 AND MonomersConfSimi_8>0.076 AND MonomersBlosum_14>0.076 AND DimersClustEm14_65< = 0.001 AND DimersClustEm14_100< = 0.002 AND TrimersNatural_6< = 0 AND TrimersClustEm14_46< = 0.003 AND DimersNatural_5< = 0.009 AND DimersClustEm14_71< = 0.004 THEN Soluble	11	49	2.04
IF Length< = 233 AND MonomersClustEm17_34< = 0.136 AND MonomersBlosum_16< = 0.173 AND DimersConfSimi_14>0.020 AND DimersClustEm14_59< = 0.040 AND MonomersClustEm17_34< = 0.113 AND TrimersNatural_0< = 0.002 AND MonomersNatural_2>0.066 AND TrimersClustEm17_80< = 0.002 AND DimersBlosum_58< = 0.009 AND DimersBlosum_38< = 0.032 AND MonomersClustEm14_22>0.022 AND DimersPhysChem_118>0.002 AND TrimersClustEm14_65< = 0.005 THEN Insoluble	14	47	2.13
**VTJ48**			
IF Length>233 AND DimersClustEm17_102< = 0.069 AND MonomersNatural_0>0.047 THEN Insoluble	3	593	17.54
IF Length< = 233 AND MonomersClustEm17_34>0.136 THEN Soluble	2	287	5.23
IF Length< = 233 AND MonomersClustEm17_34< = 0.136 AND MonomersBlosum_16< = 0.173 AND DimersClustEm14_59< = 0.040 AND MonomersBlosum_14>0.086 AND MonomersHydro_0>0.324 THEN Insoluble	6	100	30.00
IF Length< = 233 AND MonomersClustEm17_34< = 0.136 AND MonomersBlosum_16< = 0.173 AND DimersClustEm14_59< = 0.040 AND MonomersBlosum_14< = 0.086 THEN Soluble	5	99	21.21
IF Length< = 233 AND MonomersClustEm17_34< = 0.136 AND MonomersBlosum_16< = 0.173 AND DimersClustEm14_59< = 0.040 AND MonomersBlosum_14>0.086 AND MonomersHydro_0< = 0.324 AND MonomersClustEm17_34>0.110 THEN Soluble	7	68	20.59

### 3.4 User Study

**Figure 4:**
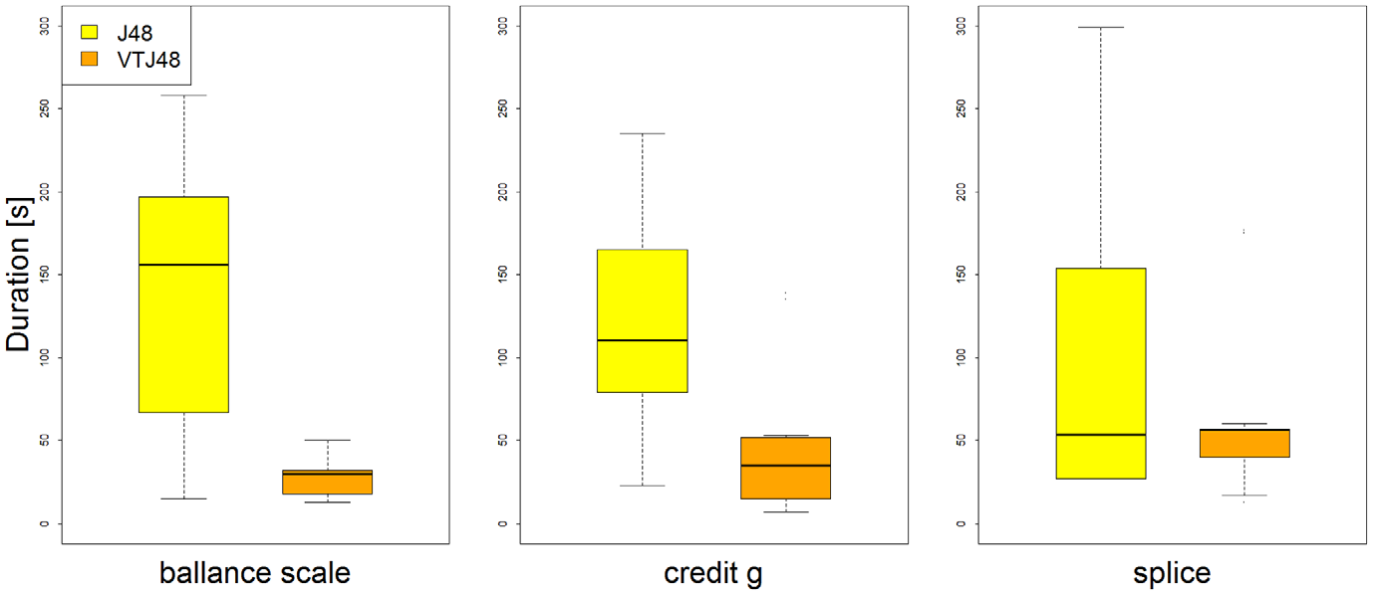
Pseudocode of decision tree reduction in Visually Tuned J48.

To test the effectiveness of the VTJ48 method in terms of usability, a Weka package was developed implementing the visually constrained tree building algorithm. An experiment to compare the duration of building decision trees using the J48 and VTJ48 Weka packages in Weka Explorer was set up. Three different datasets from the UCI repository (balance-scale, credit-g, and splice) were chosen based on their complexity where the need for tuning the tree models is more likely to be necessary. Fourteen master students, all enrolled in a Bioinformatics program, were recruited to take part in the experiment. After a brief introduction to the VTJ48 Weka package, the participants were given the datasets and were asked to build a comprehensible decision tree from each dataset using both, J48 and VTJ48 methods. Additionally, the participants were instructed to optimize each decision tree to fit to a single computer screen to allow optimal comprehensibility. In the case of J48 classifiers, this meant tuning the binary splits, minimal number of objects, and pruning parameters. In the case of VTJ48, this simply meant setting the desired resolution parameters. The duration from the start of the tree building process to the point when the decision tree was displayed on a single screen, was stored for further analysis. Figure 4 clearly shows that tree building times were shorter for VTJ48 method on all datasets.

In order to test the statistical significance of the obtained results, the Wilcoxon signed-rank test was chosen to compare the distributions of tree building times and accuracies for different tree building methods. Each of the tests was assessed at a significance level of 95%. The medians of tree building times for the J48 and VTJ48 methods were significantly different for two datasets (balance-scale: p = 0.002, credit-g: p = 0.020). Tree building times were not significantly different for the splice dataset (p = 0.396), however, the mean tree building time was still 33.14 seconds shorter for the VTJ48 method.

## Discussion

This study focused on evaluation of decision tree performance when useful and comprehensible decision trees are needed. The evaluation was done on 30 datasets from the following two areas in bioinformatics: protein solubility classification and gene expression analysis.

More precisely, strict boundaries for width and height of built decision trees were set to produce more comprehensible trees. It is important to note that VTDT approach only helps the end-user in tuning the decision tree building parameters and does not propose a novel decision tree building algorithm. Although this paper presents the automated visual tuning on C4.5 decision trees, it would be possible to adapt the VTDT principles to any other decision tree building algorithm that requires tuning of parameters to achieve optimal results. By tuning the parameters, without interfering with the internal decision tree building process and constraining the tuning only by the dimensions of the decision tree, the bias of influencing the classification performance is avoided.

The results of our study confirmed there is no statistically significant difference in predictive performance between the decision trees built using default values and the ones that were built using the proposed process of visual tuning. Moreover, when AUC is observed, visually tuned models, that are usually also much simpler than large default models, performed better on majority of datasets. This is especially true for most of the protein and gene expression datasets, where the performance improvements were significant. However, it has to be noted that a larger sample of datasets would be needed to draw more reliable conclusions. Based on these results, one could conclude that simpler models usually produce at least comparable results if not better. This has also been shown in many other studies related to the Occam's razor theory [39], [40]. However, there are also studies that demonstrate the contrary - i.e., growing the trees will improve the classification performance [41]. To sum it up, it all depends on how a simple model is defined. In the case of VTDTs, we should probably state that if the model is simple enough (i.e., fits into our predefined visual boundaries), it will produce good or even better results than most of the more complex models. Unfortunately, the proposed decision tree tuning suffers from the high time complexity in comparison to classical decision tree that is built only once. However, as shown by the the user study, it still saves a lot of time in comparison to manual tuning and fitting of the decision tree to desired dimensions.

In this paper we evaluated the visual tuning strategy only on C4.5 decision trees. From the research and also from the practical usability point of view, it would be important to extend this study and consequently develop a Weka package that would allow simultaneous tuning of different decision tree models (e.g. CART [4]). Some of the areas where visual tuning could also be applied are comprehensible ensembles of classifiers or variations of decision tree models (e.g, Alternating decision trees [42]). These models combine boosted decision stumps in a structure where visual constraints could be beneficial for the end-user in different areas of bioinformatics.
